# Effects of Normal Force on the Tribocorrosion Behavior of a Nickel-Based Superalloy in Alkaline Solution: An Electrochemical Study

**DOI:** 10.3390/ma13183959

**Published:** 2020-09-07

**Authors:** Long Xin, Liwu Jiang

**Affiliations:** National Center for Materials Service Safety, University of Science and Technology Beijing, Beijing 100083, China; long_xin@ustb.edu.cn

**Keywords:** tribocorrosion, electrochemistry, passive film, normal force

## Abstract

The tribocorrosion behavior of Inconel 690TT in NaOH (pH = 9.8) solution at different normal forces was investigated by an electrochemical method. The results indicated that normal force had a great effect on the tribocorrosion behavior and mechanism. When normal force increased from 15 to 30 N, fretting regime was in gross slip regime (GSR), and wear volume gradually increased. When normal force further increased to 45 N, wear volume significantly decreased due to the fretting regime changing from GSR to partial slip regime (PSR). When fretting ran in GSR, the corrosion resistance decreased with the negative shift of open circuit potential (OCP). However, when the fretting regime changed to PSR, the corrosion reaction significantly decreased due to the adhesive wear. Fretting wear broke the passive film at the contacting surface, which caused the worn surface to be more active and prone to corrosion. However, the broken passive film was quickly repaired in subsequent oxidation. The break and repair of passive film strongly depended on normal force. In GSR, the increase in normal force aggravated the break of passive film. In PSR, the passive film was not easy to break with a further increase of normal force.

## 1. Introduction

Inconel 690TT (thermally treated) has been widely used as a structural material for steam generator (SG) tubes in nuclear power plants due to the fact of its outstanding mechanical strength and corrosion resistance [1]. However, fretting can occur in the SG tubes, which is an important issue due to the flow-induced vibration in the pressurized water reactor (PWR) [2].

Due to the pH value of the secondary side water in the PWR being usually controlled at 9.0~10.0 [3,4], the solution can act as an electrolyte for the electrochemical corrosion of the SG tube. Combined with fretting, the mechanical removal of passive oxide film accelerated the material’s deterioration [5]. Therefore, fretting corrosion of SG tubes is a combination of two simultaneous interfacial processes: One is the mechanical removal of material in the form of wear debris. The other is the chemical degradation which can be accelerated by fretting. In recent years, many investigations on fretting corrosion of Inconel 690TT have been carried out with the fretting parameters including displacement amplitude [6,7], normal force [6,7,8], cycle number [9,10], temperature [3,11,12,13], and microstructure evolution [14,15,16,17,18]. However, the electrochemical effects are ignored under such a corrosive environment, especially the evolution of passive film.

The passive oxide layer plays an important role in wet environments which not only protects the contacting materials from corrosion but also reduces the friction coefficient and wear volume [19]. Under fretting conditions, the passive film can be destroyed to make more fresh metal exposed to the solution and accelerate the corrosion behavior [20]. Therefore, fretting corrosion is closely related to the depassivation and repassivation of passive film which can be studied using electrochemical methods [21,22,23,24]. The changes in open circuit potential (OCP) can give a semi-quantitative assessment of the corrosion regime and surface state of metal material [25] which indicates the evolution of passive film. However, only a few studies have used electrochemical methods for Inconel 690TT.

In addition, the sliding regime can be transformed by the change in the normal force accompanied by the variation of wear mechanism [26,27,28]. Meanwhile, the electrochemical corrosion behavior of passive metal can be affected by wear behavior. Therefore, the interaction between wear and corrosion can be affected by the change of passive film on the contacting surface of Inconel 690TT. In the present study, the varied normal force was applied to investigate the interaction of wear and the electrochemical corrosion behavior of Inconel 690TT in alkaline solution.

## 2. Materials and Methods

An Inconel 690TT tube was split into four sections in the axial direction and subsequently rolled into square plates with a length of 14.7 mm and a thickness of 1 mm. Then, the plates were heat-treated (715 °C/15 min) to remove the residual stress. Finally, the specimens were mechanically polished to a surface roughness of Ra ≈ 0.04 µm. Before the fretting test, all specimens were ultrasonically cleaned in ethanol and acetone. The chemical composition of Inconel 690TT is shown in Table 1.

**Figure 1:**
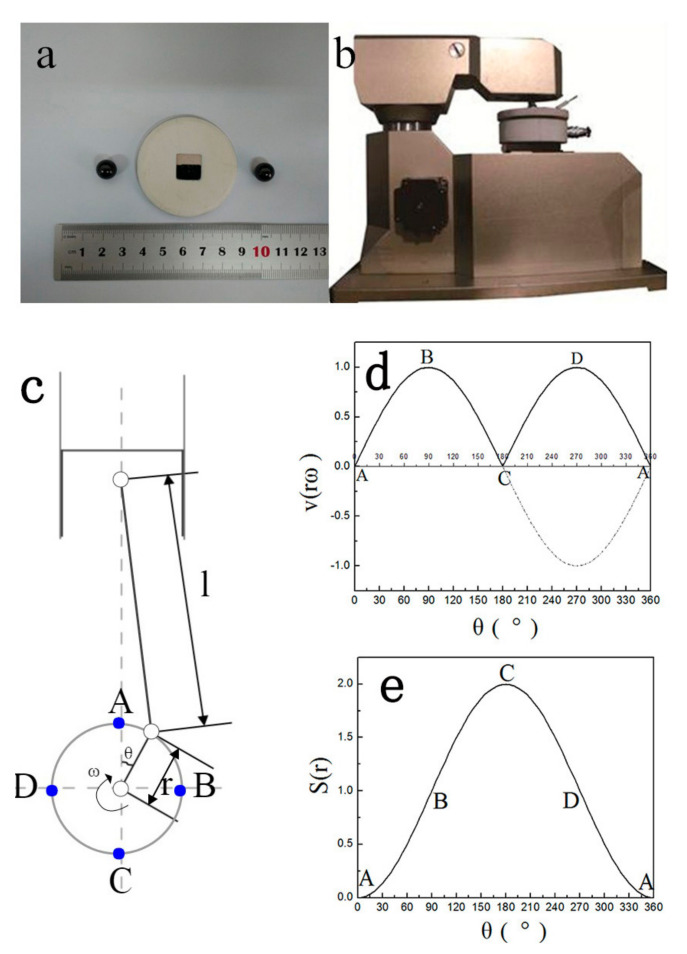
Images of (**a**) specimens and (**b**) the oscillating friction and wear tester (MFT-R4000) used in present study, along with the (**c**) schematic diagram of the crank-connecting rod in the wear tester; (**d**) the piston velocity in the crank-connecting rod; and (**e**) the piston stroke in the crank-connecting rod.

The 690TT plate was attached to the aluminum disk with a diameter of 52 mm and a thickness of 3 mm using a conductive adhesive which can ensure good electrochemical conductivity as shown in Figure 1a. The positive bare surface of the aluminum disk was sealed with liquid silicone which can provide electric insulation from aluminum disk to solution. As shown in Figure 1b, the upper ball was stable, and the lower disk was driven by the crank-connecting rod shown in Figure 1c. The formulas of the piston’s velocity and the stroke were:

(1) $$v=\sin\theta +(\frac{\lambda }{2})\sin\theta$$

(2) $$s=1-\cos\theta +(\frac{\lambda }{4})\left(1-\cos2\theta \right)$$

(3) $$\lambda =\frac{\lambda }{l}$$

Here, λ is the ratio of crank radius and connecting rod length, and its value during this test was approximately 1/500. The curves of the two formulas are shown in Figure 1d,e, respectively. The solid line in Figure 1d is the real value of the velocity which is opposite to the dashed line. From Figure 1c–e, the following conclusions can be obtained:

(1) when the crank starts rotating from point A, both the velocity and stroke values of piston are zero;
(2) when the crank rotates to point B, the stroke value becomes half of the maximum, which means the ball is at the middle way of the scratch, the velocity value of piston comes to the maximum;
(3) when the crank rotates to point C, the stroke value of piston comes to the maximum, while the velocity value becomes zero, the ball is at the other endpoint of the scratch, and the piston starts to move to the opposite direction;
(4) when the crank rotates to point D, the condition is the same to that at point B;

When the crank rotates to point A again, both values come back to zero, one cycle of fretting finishes, and a new cycle starts.

All fretting tests were carried out in NaOH (pH = 9.8) solution at a displacement amplitude of 180 µm and a frequency of 0.02 Hz. The normal force was applied at 15, 20, 30, and 45 N, respectively.

The integrated 3 electrode electrochemical workstation (CS-350) was used with the tribocorrosion tester (MFT-R4000) to monitor the electrochemical response at the contact area. The sample worked as the working electrode, the high-purity graphite worked as the counter electrode, and the saturated calomel electrode (SCE) worked as the reference electrode. The open circuit potential (OCP) and friction coefficient (FC) values were continuously recorded to quantify the fretting corrosion behavior throughout the test. After the test, the wear volume was measured by laser scanning confocal microscope (LSCM). The morphologies of wear surface were obtained by scanning electron microscope (SEM).

## 3. Results

**Figure 2:**
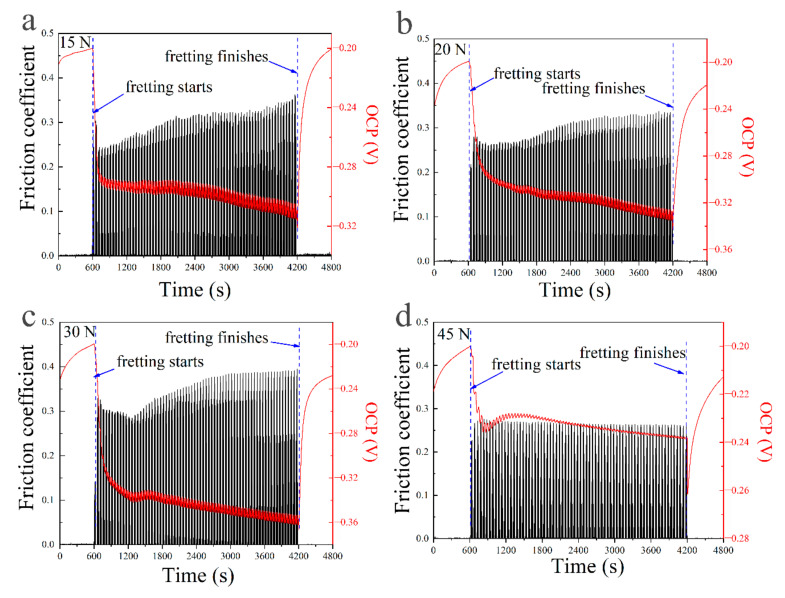
The open circuit potential (OCP) and friction coefficient (FC) curves with test time at the normal forces of (**a**) 15 N, (**b**) 20 N, (**c**) 30 N, and (**d**) 45 N.

Figure 2 shows the curves of OCP and FC at different normal forces in the fretting corrosion test. It can be found that there was a 10 min soaking time just before the fretting test. All FC curves kept to zero and the OCP curves continuously increased. Once the fretting started, all FC curves slowly increased with the test time, and their values changed in the range of 0.20~0.32. Meanwhile, all the OCP curves immediately dropped first, then decreased slowly with the test time. It is worth noticing that the OCP curves had fluctuations within a small range (0.001~0.008 V) during the fretting test. Finally, when the fretting test finished, all FC values decreased to zero immediately, and all OCP values quickly increased. 

**Figure 3:**
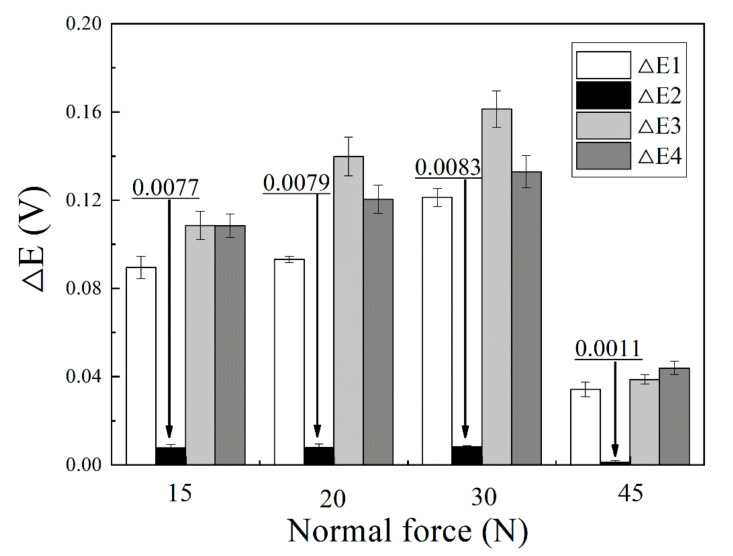
The differences in OCP for different time durations at different normal forces. The error bar was calculated by the standard deviation of three data sets.

Figure 3 shows the differences of OCP in different time durations at different normal forces. ∆E1 is the difference of OCP between 10 and 15 min, which represents the negative shift of OCP at the initial process of the fretting test. The ∆E2 is the average value of OCP fluctuation during the whole fretting test, representing the change of OCP under each fretting cycle. The ∆E3 is the difference of OCP between 10 and 70 min, which means the overall negative shift of OCP through the fretting test. The ∆E4 is the difference of OCP between 70 and 80 min, which represents the recovery of OCP after the fretting test. It can be seen that all the differences in potential increase significantly when the normal force increases from 15 to 30 N. However, when the normal force further increases to 45 N, all of them decreased significantly.

**Figure 4:**
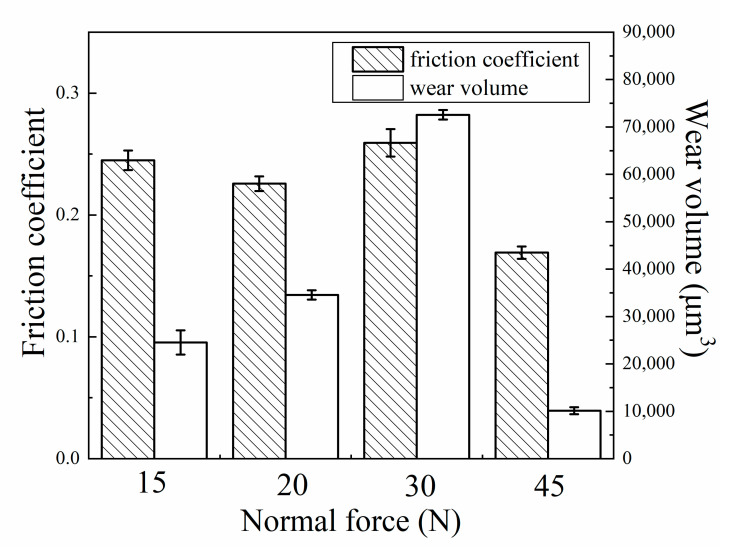
The average FC and wear volume at different normal forces. The error bar was calculated by the standard deviations of three data sets.

Figure 4 shows the average FC in the whole fretting test and the wear volume of wear scar at different normal forces. When the normal force increased from 15 to 30 N, the average value of FC exhibited a small change in the range of 0.23~0.26. When the normal force further increased to 45 N, the FC value decreased to 0.16. Correspondingly, the wear volume increased from 25,000 to 71,000 μm^3^, when the normal force increased from 15 to 30 N. Then, the wear volume sharply decreased to approximately 10,000 μm^3^, when the normal force further increased to 45 N.

**Figure 5:**
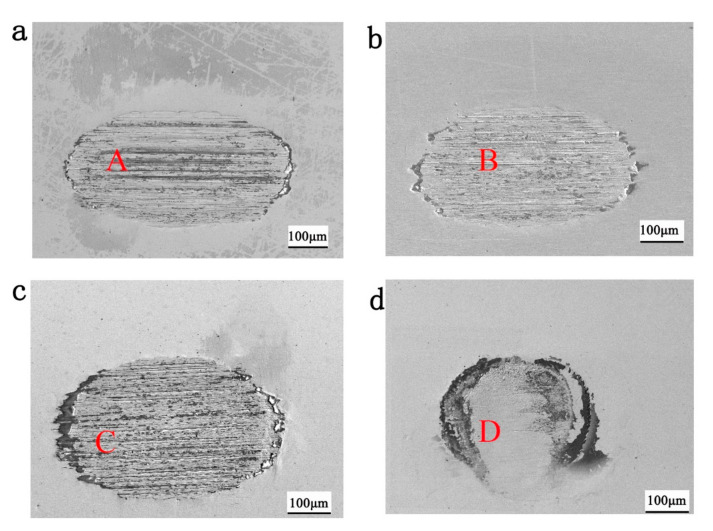
SEM images of the wear scars at normal forces of (**a**) 15 N, (**b**) 20 N, (**c**) 30 N, and (**d**) 45 N.

Figure 5 shows the SEM images of wear scars at different normal forces. It can be found that the width of the wear scar increased, while the length decreased with increasing normal force. The abrasive grooves can be found on the wear surface in Figure 5a–c. The density of the grooves increases with the increase of normal force. However, when the normal force further increased to 45 N, no abrasive groove could be found in the center of the wear scar. In addition, a dark annular region could be found surrounding the center circular area in the wear scar when the normal force was 45 N.

**Figure 6:**
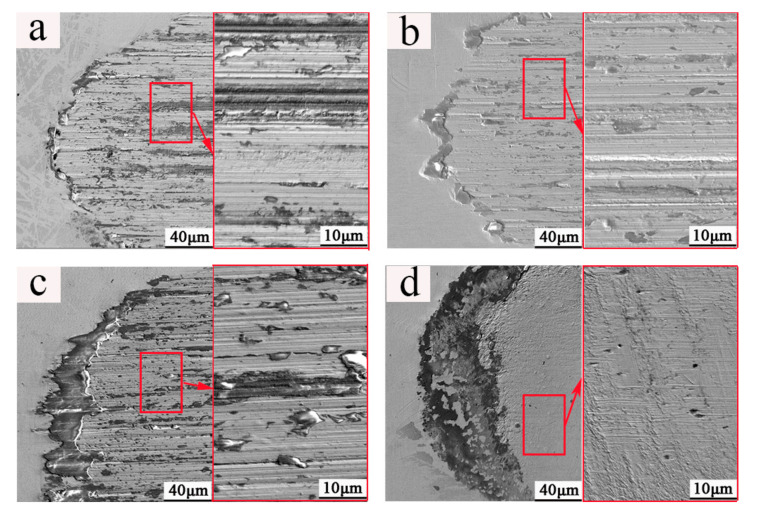
The magnified images of positions (**a**) A, (**b**) B, (**c**) C, and (**d**) D in Figure 5.

Figure 6 presents the high magnification images of marked zones in Figure 5. When the normal force increased from 15 to 30 N, many abrasive grooves could be found in the center of the wear scar as shown in magnified parts in Figure 6a–c. When the normal force further increased to 45 N, only traces of adhesive wear could be found in the center of the wear scar as shown in the magnified part in Figure 6d.

**Figure 7:**
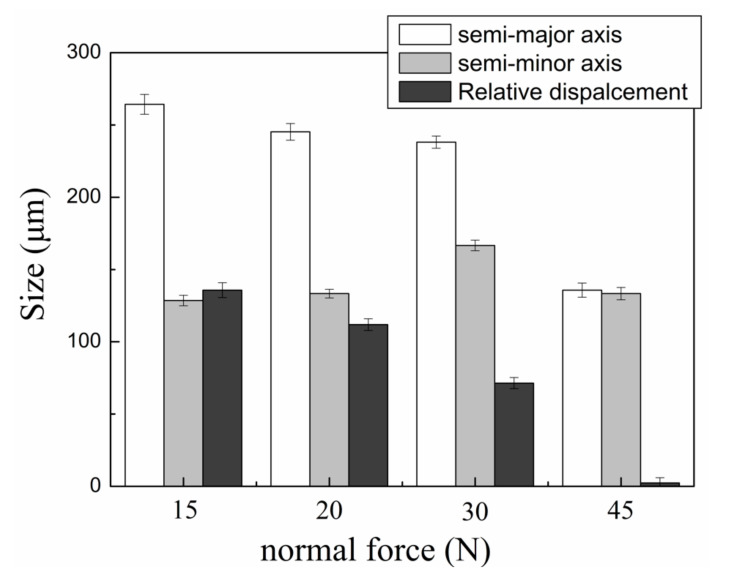
The sizes of the semi-major axis, semi-minor axis, and relative displacement of the wear scar at different normal forces. The error bars were calculated by the standard deviations of three data sets.

Figure 7 shows the size of the semi-major axis, semi-minor axis, and relative displacement of the wear scar at different normal forces. The wear scar was found as an eclipse which can be described using sizes of a semi-major axis and a semi-minor axis. The size of the semi-major axis of the wear scar always decreased with the increase of normal force. By comparison, the size of the semi-minor axis first increased when the normal increased from 15 to 30 N, then decreased when the normal force further increased to 45 N. The relative displacement of the wear scar showed a downward trend with increasing normal force. It almost became zero when the normal force was 45 N.

**Figure 8:**
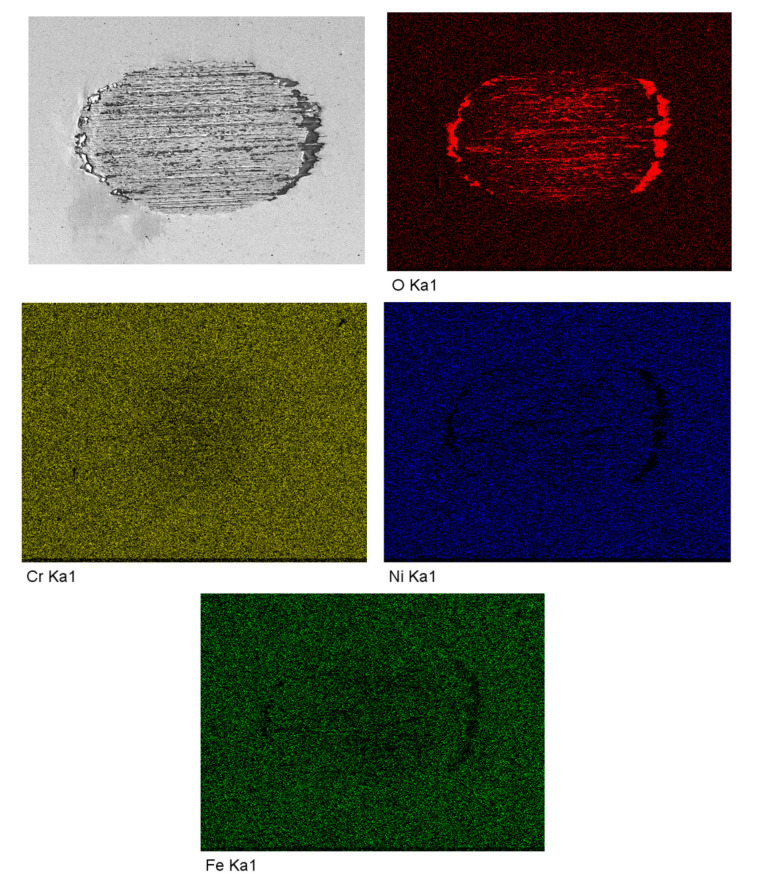
EDS mapping–scanning results of wear surface at the normal force of 30 N.

Figure 8 shows the EDS mapping–scanning results of wear surface at the normal force of 30 N. It can be found that the oxygen content on the wear surface was higher than that on the intact area, chromium content had little difference while nickel and iron contents were lower on the wear surface than that on intact surface.

**Figure 9:**
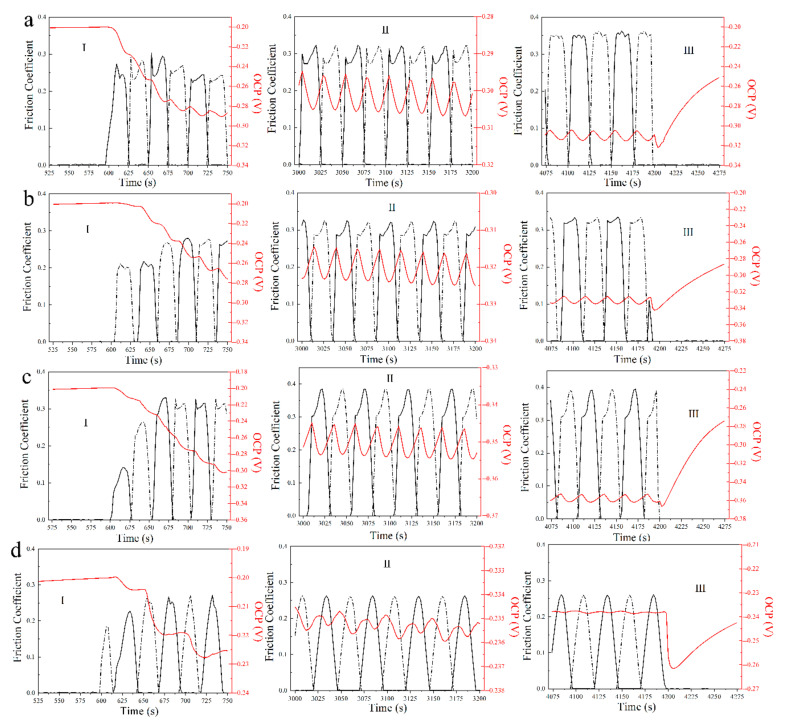
The high magnification images of part I, II, and III of the tribocorrosion curves in Figure 2 at the normal forces of (**a**) 15 N, (**b**) 20 N, (**c**) 30 N, and (**d**) 45 N.

Figure 9 shows the high magnification images of part I, II, and III of tribocorrosion curves in Figure 2. It is noted that the friction sensor can only record the values in one direction during the test, resulting in the lack of data for FC in the opposite direction which is almost the same as the recorded values. In order to analyze the data conveniently, the missing values were made up by dashed lines utilizing a simulation method. From parts I in Figure 9, it was found that when the fretting test starts, the FC curves increased immediately and showed some changes at the beginning. While the OCP curves decreased step by step. From parts II in Figure 9, it was found that in the middle of the fretting test, the FC and the OCP curves keep stable and fluctuated regularly, and the changes of the FC show a correspondence with the changes of OCP in half of one fretting cycle. After 60 min, when the load was removed, the FC curves came back to zero immediately, while the OCP curves continually increased as shown in parts III in Figure 9. However, when the normal force was 45 N, the OCP first decreased to about 0.025 V as soon as the fretting finished and increased continually. The value of the decreasing OCP was much larger than the fluctuation value of potential during the fretting test.

It is worth noting that more details can be found in the fluctuations of the OCP and FC curves during the fretting test. From Parts II in Figure 9 demonstrating the OCP and FC changes for 4 cycles obtained during the fretting tests with different normal forces, an interesting repetitive cyclic OCP change can be seen when the normal force increased from 15 to 30 N, demonstrating a potential peak around an FC peak when the fretting started and then a potential nadir around a higher FC peak. When the normal force further increased to 45 N, the potential nadir was around the only FC peak. The fluctuation of OCP and FC can be consistently seen throughout the fretting test. The time period between the nadir and the peak of the OCP was similar in all cases, which was 12 ± 1 s. The time period of one fretting cycle was 50 s; one fretting cycle contained two cyclic changes of OCP.

## 4. Discussion

It was reported that the fretting corrosion behavior was greatly affected by the mechanical factors and electrochemical corrosion [25]. The protective passive film can be broken and repaired by the interaction of wear and corrosion.

### 4.1. Surface State

The shape of the wear scar was found as an ellipse due to the slip that happened in the contacting area which was parallel to the fretting direction and larger than the contact size [29] as shown in Figure 5. When the normal force increased from 15 to 30 N, the wear scar size of the semi-major axis was larger than the size of the semi-minor axis as shown in Figure 7. The abrasive grooves were found in Figure 6, forming a typical gross slip morphology in the wear scar [16]. When the normal force increased to 45 N, the shape of the wear scar became round which was indicated by the sizes of the semi-major axis and semi-minor axis shown in Figure 7. No abrasive groove was found in the wear scar, as shown in Figure 5. In addition, the dark annular region around the wear scar was defined as s micro-slip region [30], where a micro-slip happened at the edge of interface due to the increase of shear stress and decrease of friction.

The relative displacement shown in Figure 7 was used to analyze the fretting conditions. It was found that the relative displacement decreased with the increasing normal force. When the normal force increased to 45 N, the relative displacement was almost zero, the contact pressure was high enough to prevent the relative displacement at the interface which indicated the fretting wear kept in stick-slip condition [31]. Obviously, when the normal force increased from 15 to 30 N, the fretting regime was mainly a gross slip regime; when the normal force further increased to 45 N, the fretting regime changed to a partial slip regime.

### 4.2. Wear and Corrosion Behaviors

It is proposed that the surface damage under fretting corrosion mainly resulted from mechanical wear, corrosion, and interaction of wear and corrosion. Hence, the total wear volume (*V_tot_*) can be expressed as [32]:

*V_tot_* = *V_c_* + *V_m_* + (*∆V_cm_* + *∆V_mc_*) (4)

where *V_tot_* is the total wear volume, *V_c_* is the wear volume due to the corrosion without fretting, *V_m_* is the wear volume due to the mechanics without corrosion, *∆V_cm_* is the synergistic wear volume induced by the corrosion which enhances wear due to the mechanics, and *∆V_mc_* is the synergistic wear volume induced by mechanics which enhances wear due to the corrosion. In this study, the test solution and test time were fixed, so the wear volume due to the corrosion without fretting was a constant, which would not affect the change of total volume.

When the normal force increased from 15 to 30 N, the fretting regime was changed to gross slip regime and the average friction coefficient had little changes as shown in Figure 4. Some researchers studied the influence of fretting parameters on friction coefficient and, finally, found that when the materials and test condition were established, the friction coefficient was independent on normal force under gross slip regime [33]. Therefore, in this study, the friction coefficient could be seen as a stable value. On the other hand, the surface state and the wear debris affected the wear state to some extent, and it was reasonable for the friction coefficient to have some fluctuations. 

According to the Archard model, the wear coefficient can often be extrapolated from the following relationship [33]:

(5) $$K=\frac{V}{PS}$$

where *K* is the Archard wear coefficient which strongly depends on the wear mode, displacement, and so on [25], *V* the mechanical wear volume of the fretting scar in the fretting test, *P* is the normal force, and *S* is the sliding distance which is a constant in this paper. In this paper, the Archard wear coefficient *K* can be regarded as a constant in the gross slip regime [25]. When the fretting regime was in gross slip regime without obvious elastic and plastic deformation in the wear scar, the wear volume was in a positive correlation with friction. The Archard model can be modified as:

(6) V=K′fS

where *K*’ is the modified wear coefficient, and *f* is the tangential force. In order to simplify the calculation and analysis, the tangential force *f* can be replaced by μ·P, where μ is the average friction coefficient. The wear volume induced by mechanical wear mostly with the μ can be written as follows:

(7) V=K′μPS

When the normal force increased from 15 to 30 N, the μ could be seen as a constant, so the normal force *P* was the main factor which could greatly influence the mechanical wear volume *V_m_*. As a result, with the increase of the normal force from 15 to 30 N, the *V_m_* would exhibit an upward trend just like the total wear volume shown in Figure 4. When the normal force further increased to 45 N, the fretting regime changed to a partial slip regime; the modified Archard model, which is suitable for a gross slip regime, could not be utilized in this situation [34]. Under the partial slip regime, the *V_m_* of the specimen mainly came from elastic and plastic deformation which was relatively smaller compared to that under sliding wear. As a result, the *V_m_* would show an obvious decrease in a partial slip regime. In conclusion, the mechanical wear volume increased with the increasing normal force in the gross slip regime. However, when the normal force was large enough to change the fretting regime into a partial slip regime, the mechanical wear volume decreased sharply.

The synergistic wear volume induced by corrosion enhances wear due to the mechanics and mechanics enhances wear due to the corrosion can be analyzed by the interaction of wear and corrosion. In an electrolyte, the potential that the material adopts when it is not connected to any external electrical source called open circuit potential (OCP) [35]. The anodic reaction rate is equal to the cathodic reaction rate, so the OCP can give a semi-quantitative assessment of material’s passivity and corrosion regime [35,36]. It was found that all tests showed similar changes of OCP as shown in Figure 2. When the specimens were immersed into NaOH solution for 10 min, an increasing OCP to −0.2 V was observed, suggesting the development of new protective oxide film [34]. A sharp decrease in the OCP in the range of 0.04~0.12 V was found as soon as normal force was applied, suggesting a mechanically induced depassivation of the surface which increased the corrosion rate [34]. During the fretting test, regular fluctuations of potential were found demonstrating periodically depassivation and repassivation of the wear surface. After fretting wear tests, an increase of OCP in the range of 0.04–0.13 V was found, representing continuous repassivation of the wear surface and apparent decrease in the corrosion rate [35]. In Figure 3, the negative shift of OCP represented the decrease of metal corrosion resistance. The larger the negative shift of OCP, the worse the corrosion resistance of the metal surface exhibited [35]. It is believed that the increase of wear rate induced by increasing normal force could break more passive film, thus the wear scar would become more active and the corrosion reaction would be accelerated [34]. Therefore, the corrosion rate increased with the increase of wear rate. On the other hand, when the normal force increased to 45 N, the fretting regime changed to partial slip regime, the values of ∆E1, ∆E2, and ∆E3 all decreased significantly, suggesting the mechanical wear and corrosive effect became weak. The reaction area would decrease greatly when only micro-slip happened in the surrounding partial slip regime, making a little decrease and fluctuation of potential, as shown in Figure 3. When the fretting finished, the fresh worn surface would be exposed to the solution, a great decrease of OCP took place once the load was removed, as shown in Figure 8. In addition, corrosion behavior could also accelerate the wear behavior at the same time. The corrosion reaction could make the material loose and porous, so the material could be scraped more easily which would increase the wear volume [35].

In conclusion, the promotion of wear to corrosion reaction would increase with increasing normal force in gross slip regime then decrease in partial slip regime. The change of (∆*V_cm_* + ∆*V_mc_*) would be consistent with the changes of ∆E1, ∆E2 and ∆E3 in Figure 3.

### 4.3. Changes of Passive Film

Inconel 690TT has high corrosion resistance in wet environment for its passive film formed on the surface which can provide resistance to localized corrosion [21]. The passive film on nickel-based alloys in aqueous solution was generally considered as a duplex film structure [37]. The inner layer of the chromium oxide was dense and less porous, which can protect the metal surface from electrochemical corrosion. The outer layer of the iron oxide was porous and less adhesive, which was usually formed by dissolution or precipitation mechanism. Based on References [37,38], the steady-state oxide of Inconel 690TT was FeCr_2_O_4_ at low potential in alkaline solutions. Presumably, the new oxide formed on the surface of Inconel 690TT, as shown in Figure 8, which could thicken the passive film which was a mixture of Cr_2_O_3_ and FeCr_2_O_4_ when the samples were immersed into NaOH solution for 10 min in region one as shown in Figure 2. This barrier could stop metal ions being released to the environment and make positive transition of OCP. Fretting wear tests started in region two where rapid negative potential shifts were found followed by a steady state potential evolution. The negative shift of potential was related to damage of the protective film due to fretting, and the surface became more active [32]. In region three the potential moved to the positive direction immediately after fretting wear stopped, indicating that a “recovery” of the damaged passive film occurred [25].

**Figure 10:**
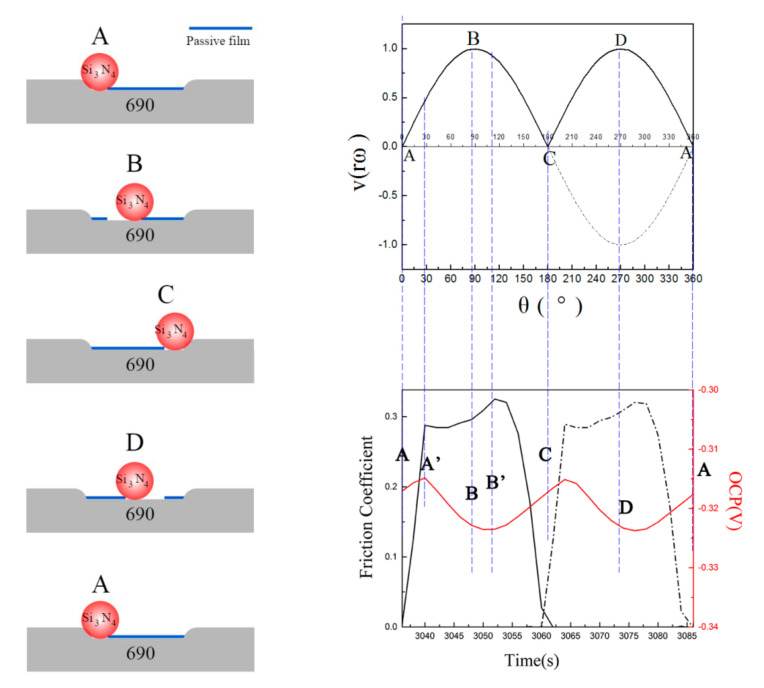
Schematic diagrams of the evolution of passive films at different normal forces during the tribocorrosion test.

From Figure 9, the OCP curves decreased step by step at the beginning of the fretting test, which indicated that the original passive film on the surface was thick enough to be removed layer by layer. During the fretting tests, original passive film on worn surface was broken by fretting wear, wear surface became more active with accelerated oxidation. Then new chromium oxide and iron oxide formed on the wear surface, which could compose a new passive film [21]. The more serious the damage of the passive film was, the more active the wear surface became. Therefore, the break of passive film could promote the corrosion reaction during the fretting wear. The broken passive film would be repaired indicated by the fluctuations of OCP as shown by the part II in Figure 9. In addition, the changes of FC showed a negative correlation to the changes of OCP in the regular fluctuations, because the passive film could reduce friction coefficient for its lubrication effect [19]. Connecting with the piston’s velocity and stroke curves shown in Figure 1c, it was proposed that there were five special points in every fretting cycle marked for the changes of the passive film in gross slip regime, as shown in Figure 10. When the fretting cycle started in point A, the ceramic ball was in the left endpoint of the wear scar, the velocity of the piston was zero, the potential was near to the peak while the FC was zero. The FC increased to a peak rapidly in a few seconds while the potential was at the peak. The relative fretting motion between the ball and plate started at this point A’ due to the existence of static friction [39]. The crank kept rotating and the velocity of piston increased to the maximum in point B, where the ceramic ball should be in the midpoint of the wear scar. From point A’ to B, the FC had an upward trend on the whole, while the OCP had a downward trend. In this progress, the damage of the passive film on the contact area dominated with the increase of the piston’s velocity and the lubrication effect of passive film reduced, which led to a negative shift of OCP and increase of FC. Then the fretting motion kept going, the piston’s velocity decreased to zero at point C, where the ceramic ball should be in the endpoint of the wear scar. From point B to C, the FC first increased then decreased to zero, while the OCP first had a negative shift then a positive shift. In this process, the oxidation behavior was enhanced by the former mechanical wear, the repair of the passive film dominated with the decrease of the piston’s velocity, which led to a positive shift of the OCP. It is worth noting that the peak of FC curve occurred just at the nadir the OCP curve marked as point B’ close to point B. This phenomenon should happen at point B, where the damage extent of the passive film is at a maximum theoretically. The reasons for this delay phenomenon might be the elastic deformation, influence of wear debris, and sensitivity of the machines. Cyclic fretting motion in one direction finished in point C, the values of OCP and FC were the same to those in point A, and the situations happened in the opposite direction. The break and repair of passive film dominated repeatedly and mutually during the whole fretting test. However, when the fretting regime changed to a partial slip regime, no slip happened at the interface barely. The break and repair of passive film only happened in the micro-slip region, which is much smaller than the adhesive region; therefore, the fluctuation of potential would be much smaller compared to those at lower normal forces as shown in Figure 3.

## 5. Conclusions

In this study, the tribocorrosion behavior of Inconel 690TT in alkaline solution at different normal forces was investigated. The FC and OCP were obtained by in situ electrochemical measurements. The main conclusions are as follows:

1. The normal force had a great effect on the tribocorrosion behavior and mechanism. When the normal force increased from 15 to 30 N, the fretting regime was in a gross slip regime and the wear volume had an upward trend. When the normal force further increased to 45 N, the wear volume decreased significantly due to the change in the fretting regime from a gross slip regime to a partial slip regime;
2. The electrochemical behavior on the worn surface strongly depended on the fretting wear mechanism. The fretting wear accelerated the corrosion reaction at the contact area. When fretting ran in a gross slip regime, the corrosion resistance decreased with the increase of normal force by negative shifts of OCP. However, when the fretting regime changed to a partial slip regime, the corrosion reaction decreased significantly due to the adhesive wear;
3. Passive film played an important role in the fretting corrosion behavior. Fretting wear broke the passive film at contacting surface, which caused the worn surface to be more active and more prone to be corrosive. However, the broken passive film was quickly repaired by oxides produced in subsequent oxidation, and the corrosion continued to occur only after the break of passive film by the fretting wear;
4. The break and repair of passive film on the wear scar strongly depended on the normal force. In the gross slip regime, the increase of normal force aggravated the break of passive film. In the case of a partial slip regime, the passive film was as easily broken with a further increase in the normal force.

## Figures and Tables

**Table 1:** Chemical composition of Inconel 690TT (wt.%).

Specimen	Element								
	Ni	Fe	Cr	C	Ti	Mn	Si	P	S
Inconel 690TT	Bal	11.6	29.9	0.025	0.30	0.25	0.33	0.086	0.0025
